# Effect of the fluid management nursing intervention on improving biochemical test results and dialysis therapy in chronic kidney disease patients: a randomized controlled trial

**DOI:** 10.17533/udea.iee.v43n3e12

**Published:** 2025-11-06

**Authors:** José Erivelton de Souza Maciel Ferreira, Daniel Freire de Sousa, Rafaella Pessoa Moreira, Huana Carolina Cândido Morais, Lívia Moreira Barros, Tahissa Frota Cavalcante

**Affiliations:** 1 Research Nurse, Ph.D. student. Email: eriveltonsmf@gmail.com. Corresponding author. https://orcid.org/0000-0003-2668-7587 Universidade da Integração Internacional da Lusofonia Afro-Brasileira Brazil eriveltonsmf@gmail.com; 2 Research Pharmacist, Ph.D. Professor. Email: daniel@unilab.edu.br https://orcid.org/0000-0002-2333-5343 Universidade da Integração Internacional da Lusofonia Afro-Brasileira Brazil daniel@unilab.edu.br; 3 Research Nurse, Ph.D. Professor. Email: rafaellapessoa@unilab.edu.br https://orcid.org/0000-0003-2341-7936 Universidade da Integração Internacional da Lusofonia Afro-Brasileira Brazil rafaellapessoa@unilab.edu.br; 4 Research Nurse, Ph.D. Professor. Email: huanacarolina@unilab.edu.br https://orcid.org/0000-0001-6435-1457 Universidade da Integração Internacional da Lusofonia Afro-Brasileira Brazil huanacarolina@unilab.edu.br; 5 Research Nurse, Ph.D. Professor. Email: livia@unilab.edu.br https://orcid.org/0000-0002-9763-280X Universidade da Integração Internacional da Lusofonia Afro-Brasileira Brazil livia@unilab.edu.br; 6 Research Nurse, Ph.D. Professor. Email: tahissa@unilab.edu.br https://orcid.org/0000-0002-7758-4273 Universidade da Integração Internacional da Lusofonia Afro-Brasileira Brazil tahissa@unilab.edu.br; 7 University of International Integration of Afro-Brazilian Lusofonia, Redenção ( Ceará), Brazil. Universidade da Integração Internacional da Lusofonia Afro-Brasileira University of International Integration of Afro-Brazilian Lusofonia Redenção Ceará Brazil

**Keywords:** renal insufficiency chronic, renal dialysis, water-electrolyte balance, nursing care., insuficiencia renal crónica, diálisis renal, equilibrio hidroelectrolítico, atención de enfermería., insuficiência renal crônica, diálise renal, equilíbrio hidroeletrolítico, cuidados de enfermagem.

## Abstract

**Objetive.:**

To test the effectiveness of nursing intervention to control fluid volume on improving laboratory test results and dialysis adequacy in patients with Excess fluid volume.

**Methods.:**

This is a randomized, double-blind, parallel-group controlled trial involving 34 patients with chronic kidney disease and a nursing diagnosis of Excess Fluid Volume undergoing chronic hemodialysis equally randomized into two groups (control *n*=17 and intervention *n*=17). Data were collected on sociodemographic and clinical factors, the presence of Excess Fluid Volume, and water balance. Laboratory parameters, including serum electrolytes, urea, creatinine, and dialysis adequacy markers, were assessed before and after the intervention. The intervention consisted of 13 nursing activities, including educational, follow-up, and reminder components, such as fluid balance monitoring, daily weight control, edema assessment, laboratory follow-up, and health education on diet and self-care. The control group received only the usual care provided at the dialysis clinic.

**Results.:**

There were significant improvements in laboratory test results and dialysis adequacy. The statistical difference between the groups was significant in the mean values of calcium (*p<*0.001), post-hemodialysis urea (*p=*0.002), and creatinine (*p=*0.006), demonstrating the direct effect of the intervention. In addition, there were improvements in overall dialysis quality and adequacy measures.

**Conclusion.:**

The nursing intervention significantly improved laboratory test results and dialysis adequacy in patients with chronic renal failure and Excess Fluid Volume, highlighting its potential for enhancing patient management and nursing care.

## Resumen

**Objetivo.:**

Evaluar la eficacia de la intervención de enfermería para controlar el volumen de líquidos en la mejoría de los resultados de las pruebas de laboratorio y en la adecuación de la diálisis en pacientes con exceso de volumen de líquidos**.**

**Métodos.:**

Se trata de un ensayo aleatorio, doble ciego, controlado, de grupos paralelos, en el que participaron 34 pacientes con enfermedad renal crónica y un diagnóstico de enfermería de exceso de volumen de líquidos sometidos a hemodiálisis crónica, aleatorizados por igual en dos grupos (control *n*=17 e intervención *n*=17). Se recopilaron datos sobre factores sociodemográficos y clínicos, la presencia de exceso de volumen de líquidos y del equilibrio hídrico. Se evaluaron los parámetros de laboratorio, incluidos los electrolitos séricos, la urea, la creatinina y los marcadores de adecuación de la diálisis, antes y después de la intervención. La intervención consistió en 13 actividades de enfermería, que incluían componentes educativos, de seguimiento y de recordatorio, como la monitorización del equilibrio hídrico, el control diario del peso, la evaluación del edema, el seguimiento de laboratorio y la educación sanitaria sobre la dieta y el autocuidado. El grupo control solo recibió la atención habitual que se proporciona en la clínica de diálisis.

**Resultados.:**

Se observaron mejorías significativas en los resultados de las pruebas de laboratorio y en la adecuación de la diálisis. La diferencia estadística entre los grupos fue significativa en los valores medios de calcio (*p*<0.001), urea post-hemodiálisis (*p*=0.002) y creatinina (*p*=0.006), lo que demuestra el efecto directo de la intervención. Además, se observaron mejorías en la calidad general de la diálisis y en las medidas de adecuación.

**Conclusión.:**

La intervención de enfermería mejoró significativamente los resultados de las pruebas de laboratorio y la adecuación de la diálisis en pacientes con insuficiencia renal crónica y exceso de volumen de líquido, lo que pone de relieve su potencial para mejorar el manejo de los pacientes y la atención de enfermería.

## Resumo

**Objetivo.:**

Testar a eficácia de uma intervenção de enfermagem para o controle do volume de líquidos na melhora dos exames laboratoriais e da adequação da diálise em pacientes com diagnóstico de Enfermagem de Excesso de volume de líquidos.

**Métodos.:**

Trata-se de um ensaio clínico randomizado, duplo-cego, controlado, em grupos paralelos, envolvendo 34 pacientes com doença renal crônica e com a presença do diagnóstico de Enfermagem Excesso de volume de líquidos, em hemodiálise crônica, igualmente randomizados em dois grupos (controle *n*=17 e intervenção *n*=17). Foram coletados dados sociodemográficos e clínicos, a presença de Excesso de volume de líquidos e equilíbrio hídrico. Os parâmetros laboratoriais avaliados antes e após a intervenção incluíram eletrólitos séricos, ureia, creatinina e marcadores de adequação da diálise. A intervenção consistiu em 13 atividades de enfermagem, incluindo componentes educativos, de acompanhamento e de lembrete, tais como monitoramento do balanço hídrico, controle diário de peso, avaliação de edema, acompanhamento laboratorial e educação em saúde sobre dieta e autocuidado. O grupo controle recebeu apenas o cuidado usual prestado na clínica de diálise.

**Resultados.:**

Houve melhora significativa nos exames laboratoriais e na adequação da diálise. A diferença estatística entre os grupos foi significativa nos valores médios de cálcio (*p*<0.001), ureia pós-hemodiálise (*p*=0.002) e creatinina (*p*=0.006), demonstrando o efeito direto da intervenção. Além disso, observaram-se melhorias na qualidade global e nas medidas de adequação da diálise.

**Conclusão.:**

A intervenção de enfermagem melhorou significativamente os exames laboratoriais e a adequação da diálise em pacientes com insuficiência renal crônica e Excesso de volume de líquidos, destacando seu potencial para aprimorar o manejo clínico e o cuidado de enfermagem.

## Introduction

The progressive decline in glomerular filtration rate (GFR) is a hallmark of chronic kidney disease (CKD), initially evidenced by the persistent rise in plasma levels of substances normally excreted by the kidneys, such as urea and creatinine.^1^ This progressive decline compromises the body's ability to eliminate toxic substances, resulting in a variety of biochemical disorders and potential complications arising from their accumulation in the body, such as imbalances in calcium, phosphorus, potassium, sodium, and magnesium, among others.^2^ Electrolyte imbalance in this population can lead to water overload, serious cardiac complications, muscle weakness, neurological and renal problems, worsening their prognosis.^3^ The risk factors for mortality in chronic hemodialysis patients are not well understood, but there is a positive association between hypokalemia, hyponatremia, and hyperphosphatemia and a significant risk of mortality.^4^ Therefore, electrolyte shifts are among the most important factors in hemodialysis patients.^5^

Hemodialysis therapy plays a critical role in the regulation of these electrolytes but is directly related to the challenge of controlling Excess fluid volume (EFV) in these patients, which can lead to greater hemodynamic instability.^6^ In this context, nurses play an essential role in maintaining adequate electrolyte and laboratory balance, assuming responsibilities that range from direct assistance during hemodialysis therapy to educating and guiding patients on various aspects of self-care and management of the individual's renal and general condition.^7^ The interventions envisaged by nurses in this context, especially those supported by the Nursing Interventions Classification (NIC), not only have the potential to improve the results of biochemical and laboratory tests in patients with end-stage CKD, but also aim to significantly improve the patient's quality of life and safety.^8^ This goal will be achieved through precise control of fluid and electrolyte balance, careful evaluation of clinical observation and analysis of laboratory and biochemical test results, and measurement of dialysis adequacy for these patients.^9^

There is a clear and urgent need for effective management of laboratory abnormalities in patients with CKD and EFV because of their significant impact on the quality of life and prognosis of these patients. The current literature presents research on the importance of volume control and dialysis adequacy, as well as studies demonstrating the relationship between electrolyte imbalances and complications in patients with CKD.^3^^,^^8^^,^^9^ However, there is a gap in specific nursing interventions focused on optimizing care and reducing the risk of complications associated with biochemical changes and inadequate dialysis. Most research focuses on medical or technological interventions, leaving the critical role of nursing in the management of these patients in the background. 

Although fluid intake is not a primary factor in altering laboratory values, its analysis remains relevant for monitoring physiological status, identifying potential variations in plasma osmolality, electrolytes, and renal function. Additionally, it helps detect unexpected side effects, such as fluid overload or dehydration, and allows for correlations with other clinical indicators that may influence the body's response. Furthermore, it ensures the safety of the intervention by preventing physiological imbalances and providing complementary data for a comprehensive understanding of its effects. Therefore, the aim of this study was to test the effect of the nursing intervention Fluid Volume Control on the improvement of laboratory tests and dialysis adequacy in patients with excess fluid volume. This research fills the identified gap and contributes to the literature with evidence on the effectiveness of nursing interventions in this context.

## Methods

Design. This is a randomized controlled trial (RCT), with two parallel groups, conducted in a dialysis center in Brazil from August 2022 to May 2023. The study followed the Consolidated Standards of Reporting Trials (CONSORT) guidelines. The study was also registered in the Brazilian Clinical Trials Registry (REBEC) - RBR-2DD6X6R.

Sample and recruitment. The population of this study was made up of patients with chronic renal failure undergoing hemodialysis in a dialysis center that serves approximately 140 patients from 13 cities, with 3 shifts, every day of the week. The population was defined as finite and relatively small. All patients were screened, and those meeting the inclusion criteria were invited to participate. No formal sample size calculation was conducted; instead, a convenience sample comprising all eligible patients from this finite population was adopted. A total of 34 patients were included in the final sample and randomized into two groups: Intervention Group (IG, *n* = 17) and Control Group (CG, *n* = 17). The inclusion criteria for the study were: a medical diagnosis of chronic kidney disease, undergoing hemodialysis treatment for a minimum of 8 months, enrollment and follow-up at the designated dialysis clinic, and being of legal age (>18 years). For participants in the RCT, the inclusion criteria were further specified as follows: a medical diagnosis of chronic kidney disease, undergoing hemodialysis for at least 8 months, age >18 years, a nursing diagnosis of excess fluid volume^10^ at baseline, and scoring less than 4 points on the average indicators of the Nursing Outcomes Classification (NOC) scale for water balance^11^ at baseline. Exclusion criteria included any condition impairing mental capacity to understand and cooperate with the study, assessed using the Mini-Mental State Examination.^12^ Withdrawal criteria encompassed patient death, transfer to another dialysis clinic, hospitalization during the study period, missing a hemodialysis session on the day of intervention or outcome assessment, kidney transplantation, discharge due to improved renal function, or voluntary withdrawal at any point during the study. 

Randomization. Randomization was conducted by an independent staff member, not otherwise involved in the study, using the Research Randomizer application. The allocation sequence was generated with simple 1:1 randomization, without blocks or restrictions. Although participants were aware of their group allocation due to the nature of the intervention, the trial adopted a double-blind design since both outcome assessors (laboratory staff) and the statistician were blinded to group assignment. Clinical staff at the dialysis center were also not informed of which patients belonged to each group. Both groups received the same routine dialysis care, with the structured nursing protocol being the only additional component for the intervention group.

Variables. The main explanatory variables assessed in the study included the sociodemographic and clinical characteristics of the participants, serving as both predictors and prognostic variables. It is important to emphasize that in RCTs, the intervention under investigation should be the sole variable differing between groups. Biochemical test values for each participant were obtained directly from the dialysis center's laboratory and biochemical test software. These values were collected both before and after the intervention in both groups to ensure accurate and consistent measurement of biochemical outcomes. The outcome variables were: 1) the change in results of biochemical tests related to fluid volume and hemodialysis (pre-hemodialysis urea, post-hemodialysis urea, creatinine, potassium, phosphorus, calcium, hemoglobin and hematocrit) between patients in the intervention group and those in the control group; 2) the change in dialysis adequacy measures (URR - Urea Reduction Rate; and Kt/V - used to assess the adequacy of hemodialysis treatment, where it reads: Dialyzer urea clearance multiplied by dialysis time divided by the volume of urea distribution, which is approximately equal to the patient's total body water). Both results were observed in both groups. These biochemical tests were selected because they have been reported to be relevant for analyzing the quality of hemodialysis in patients with CKD and because they are widely prescribed for analysis in this population.^13^ Therefore, these electrolytes were used to evaluate the effect of the proposed intervention on improving their results and dialysis adequacy. The use of furosemide was considered as a possible confounding variable. The data analyzed showed that this medication was present in 23.5% of the control group and 35.3% of the intervention group, but this difference was not statistically significant (*p=*0.708). Nevertheless, we decided to use linear regression to adjust the risk estimates to account for this confounding variable and its possible relationship with the outcome variables.

Instrument and outcome measures. Four instruments were used to collect data. The first allowed the researcher to monitor and control the process of selecting participants, as well as obtaining their sociodemographic and clinical data. The second aimed to identify the presence of excess fluid volume. The third allowed the measurement of water balance. Finally, the fourth instrument refers to the 13 groups of activities that make up the intervention carried out for the intervention group. The full version of the instrument can be found in the study by Azevedo *et al.*^6^

Data collection and research protocol. The research team consisted of members of a research group on chronic non-communicable diseases, including senior nursing students, clinical nurses, and nursing researchers with master’s and doctoral degrees. The lead researcher coordinated the process and developed the clinical cases used in the simulations. The intervention group received the structured nursing protocol in addition to routine dialysis care, whereas the control group received only the routine care usually provided at the clinic. This was a IV phases study: 

*Phase I - Baseline*, the research was presented, and participants were recruited. The 139 patients (total population) were screened to select a significant target population that met the defined inclusion criteria. Three months were dedicated to this phase, with recruitment taking place in the clinic itself. Patients who agreed to participate were taken individually to a private room where they underwent a nursing consultation to verify the inclusion and exclusion criteria. 

*Phase II - Reassessment*, there was a one-month reassessment of the subjects potentially selected to make up the research sample. This phase was considered necessary because of the mutability of human responses, which can worsen, become chronic, or even disappear. After the reassessment of the patients, the clinical indicators to be compared with the outcome were evaluated. 

*Phase III* - *the intervention* is applied to the IG. This phase lasted one month and took place in the health center itself, before the hemodialysis session. The intervention was carried out through a nursing consultation, following the protocol of Azevedo *et al.*^6^ with the aim of achieving and maintaining good biochemical results, as well as an excellent measure of dialysis adequacy, through an adequate water balance in people undergoing hemodialysis. The intervention comprised 13 distinct activities aimed at managing fluid volume: (1) Daily weighing and trend monitoring, (2) Keeping accurate records of intake and output, (3) Monitoring laboratory results for fluid retention, (4) Monitoring hemodynamic status, including mean arterial pressure, (5) Monitoring vital signs, as appropriate, (6) Monitoring indicators of fluid excess/retention, as appropriate, (7) Monitoring the patient's weight change before and after dialysis, as appropriate, (8) Assessing the location and extent of edema, if present, (9) Monitoring food/fluid intake and calculating daily caloric intake, if appropriate, (10) Advising the patient on fasting for laboratory and biochemical tests, if appropriate, (11) Distributing fluid intake over 24 hours, if appropriate, (12) Advising family members and/or caregivers on assisting the patient with eating, if appropriate, and (13) Alerting the medical team to cases of worsening signs and symptoms of fluid overload. The implementation of the different groups of activities in the intervention required the adoption or development of different tools. For the activities in groups 1 and 7, a diary was developed to allow participants to record their daily weigh-ins. For groups 2, 6 and 11, a diary was developed to record daily intake and excretion. In addition, an illustrated e-book developed by a team of nephrologist nutritionists was used to provide nutritional guidance.^14^ For the activities in groups 3, 10 and 13, the monthly laboratory tests collected and provided by the dialysis clinic were used. For the activity "Conduct health education activities on nutrition during the hemodialysis session", an educational intervention was conducted using a quiz format. This interactive educational action took place during the hemodialysis sessions with participants in the intervention group. The quiz questions and the topics discussed were based on the literature consulted in narrative form.^14^^,^^15^ All the activities described in this intervention, both those that contribute directly and those that contribute indirectly to the achievement of the proposed objective, were fully implemented. The last-mentioned tools did not require validation or analysis, as they were simple daily records of weight and intake/excretion. However, all supplementary materials were discussed by members of a research group specializing in chronic non-communicable diseases, composed of postdoctoral fellows, physicians, masters, master’s students, and undergraduate nursing students. Routine interventions in the dialysis clinic included various activities, such as daily bedside visits by the physician and nurse, weekly multidisciplinary visits involving the physician, nurse, dietician, psychologist, and social worker, and office consultations in case of significant changes in laboratory and/or imaging tests. All professionals on the unit provided regular instruction on fluid and electrolyte control, arteriovenous fistula maintenance, and general catheter care as part of their work routines. 

*Phase IV - outcomes evaluation*: semiological evaluation was conducted at the health facility prior to the hemodialysis session, and laboratory tests were performed both before and after the session. Participants in the intervention group were informed that their involvement in the research would occur at two additional points after the baseline and reassessment: the first point focused on the application of the proposed intervention, and the second point involved verifying the study's outcomes. Participants in the control group were informed that their contribution to the study would only occur at two points: at the baseline and at the final review of the study outcomes.

Data analysis. The analyses encompassed comparisons both within each group (intra-group) and between groups (inter-group). Between-group comparisons utilized Fisher's exact test for qualitative variables and the Mann-Whitney test for quantitative variables due to the nonparametric nature of the data and the small sample size. Within-group comparisons employed McNemar's test for qualitative variables and Wilcoxon's test for quantitative variables. Furthermore, linear regression was employed to assess group effects on numerical variables, with consideration given to furosemide usage as a potential confounder. All statistical analyses were conducted at a significance level of α=5%, with associations deemed significant at *p<*0.05. Stata software version 13 facilitated these analytical procedures, enabling a thorough evaluation of the intervention's impact on study outcomes.

Ethical considerations. The research strictly complied with ethical and legal principles, was approved by the Institutional Research Ethics Committee for studies involving human subjects under registration number CAAE 56761422.6.0000.5576, and all participants signed a written informed consent form.

## Results

Recruitment and retention

**Figure 1:**
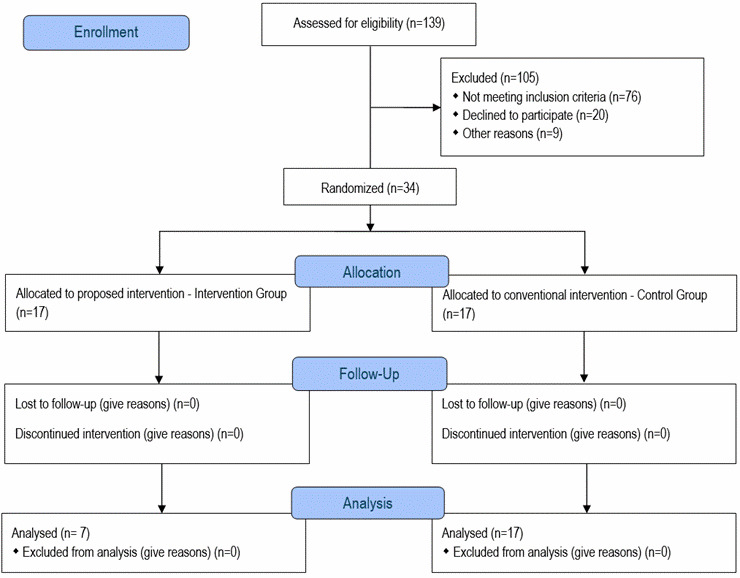
Flowchart for a detailed understanding of the procedures adopted for the execution of the randomized clinical trial.

All subjects in the study population (n=139) were examined as shown in Figure 1. From the initial baseline of 92 subjects, 22 were excluded for lack of EFV^10^, 20 for water balance >4 points on the NOC scale^11^, 9 for both lack of EFV and water balance >4 points on the NOC scale, 2 died before the intervention, and 2 were discharged due to successful transplantation and improved renal function, leaving 37 subjects. After reassessment, 1 was transferred and 2 withdrew, resulting in their exclusion from the study. After applying the inclusion, exclusion, discontinuation, and dropout criteria, 34 individuals were included in the study.

### Sociodemographic and clinical characterization of the sample.

The sociodemographic and clinical characteristics of the study population are summarized in Table 1. In this study, women predominated (61.8%), and the mean age of participants was 61.7 years. Most were married (55.9%; n = 19), and 97.1% (n = 33) underwent three hemodialysis sessions per week, with 52.9% (n = 18) having sessions of four hours or more. Control of underlying medical conditions was considered adequate in 61.8% (n = 21), and adherence to treatment was reported by 82.4% (n = 28). In all cases, there was similarity between the control and intervention groups, as *p>*0.05.

Regarding comorbidities, 41.2% (n = 14) had diabetes mellitus, 76.5% (n = 26) had systemic arterial hypertension, and 58.8% (n = 20) had heart failure. In terms of pharmacological treatment, 85.3% (n = 29) used erythropoietin, 73.5% (n = 25) received intravenous iron, 67.6% (n = 23) used folic acid, 64.7% (n = 22) used vitamin B-complex, and 50% (n = 17) used sevelamer hydrochloride. Furosemide was used by 29.4% (n = 10) of participants, with no significant difference between groups (p = 0.708). In general, there was no significant difference in the distribution of medications (p > 0.05), except for carvedilol 25 mg: 26.5% (n = 9) of participants used this drug, with one in the control group and eight in the intervention group, showing a statistically significant difference (p = 0.017). Despite this, carvedilol use did not influence the outcomes of this study.

**Table 1:** Distribution of participants in the intervention group and control group by sociodemographic and clinical variables.

Variables	Total (n=34)	CG (n=17)	IG (n=17)	p-value
Sex; n (%)				0.500^a^
Male	21 (61.8)	11 (64.7)	10 (58.8)	
Female	13 (38.2)	6 (35.3)	7 (41.2)	
Age; Mean ± SD	61.7±12.2	64.4±9.8	59.1±14.0	0.228^b^
Marital status; n (%)				0.657^a^
Single	9 (26.5)	3 (17.6)	6 (35.3)	
Married	19 (55.9)	10 (58.8)	9 (52.9)	
Widower	3 (8.8)	2 (11.8)	1 (5.9)	
Divorced	3 (8.8)	2 (11.8)	1 (5.9)	
Ethnicity; n (%)				0.349^a^
White	6 (17.6)	2 (11.8)	4 (23.5)	
Black	5 (14.7)	4 (23.5)	1 (5.9)	
Mixed	23 (67.7)	11 (64.7)	12 (70.1)	
Education; n (%)				0.135^a^
No formal education	9 (26.5)	7 (41.2)	2 (11.8)	
Incomplete primary school	16 (47.1)	8 (47.0)	8 (47.0)	
Complete primary school	4 (11.8)	1 (5.9)	3 (17.7)	
Complete high school	5 (14.7)	1 (5.9)	4 (23.5)	
Religion; n (%)				1.000^a^
Affiliated	32 (94.1)	16 (94.1)	16 (94.1)	
Unaffiliated	2 (5.9)	1 (5.9)	1 (5.9)	
Living arrangement; n (%)				0.539^a^
Alone	2 (5.9)	1 (5.9)	1 (5.9)	
With a partner	9 (26.5)	3 (17.7)	6 (35.9)	
With family	22 (64.7)	13 (76.5)	9 (52.9)	
Friends and/or acquaintances	1 (2.9)	0 (0.0)	1 (5.9)	
Employment status; n (%)				0.227
Employed	2 (5.9)	1 (5.9)	1 (5.9)	
Unemployed	3 (8.8)	0 (0.0)	3 (17.6)	
Retired	29 (85.3)	16 (94.1)	13 (76.5)	
Monthly income				0.773^b^
Mean	1513.9±773	1464.9±533.4	1562.8±972.2	
Number of hemodialysis sessions; n (%)				1.000^a^
Three	33 (97.1)	17 (100.0)	16 (94.1)	
Four	1 (2.9)	0 (0.0)	1 (5.9)	
Duration of sessions; n (%)				0.732^a^
2 to 4 hours	16 (47.1)	7 (41.2)	9 (52.9)	
>4 hours	18 (52.9)	10 (58.8)	8 (47.1)	
Control of underlying diseases; n (%)				0.481^a^
Adequate	21 (61.8)	12 (70.6)	9 (52.9)	
Slightly adequate	12 (35.3)	5 (29.4)	7 (41.2)	
Not adequate	1 (2.9)	0 (0.0)	1 (5.9)	
Knowledge about chronic kidney disease (CKD); n (%)				0.169^a^
Adequate	16 (47.1)	7 (41.2)	9 (52.9)	
Slightly adequate	14 (41.2)	6 (35.3)	8 (47.1)	
Not adequate	4 (11.8)	4 (23.5)	0 (0.0)	
Treatment adherence; n (%)				0.794^a^
Adequate	28 (82.4)	15 (88.2)	13 (76.5)	
Slightly adequate	4 (11.8)	1 (5.9)	3 (17.7)	
Not adequate	2 (5.9)	1 (5.9)	1 (5.9)	
Percentage of post-hemodialysis weight loss; n (%)				0.141^a^
8-14% loss	1 (2.9)	1 (5.9)	0 (0.0)	
5-8% loss	11 (32.4)	3 (17.7)	8 (47.1)	
1-4% loss	21 (61.8)	12 (70.6)	9 (52.9)	
No change	1 (2.9)	1 (5.9)	0 (0.0)	
Depression; n (%)				0.656^a^
Present	6 (17.7)	4 (23.5)	2 (11.8)	
Absent	28 (82.3)	13 (76.5)	15 (88.2)	
Transplanted; n (%)				0.259^a^
Yes	1 (2.9)	1 (5.9)	0 (0.0)	
No	33 (97.1)	16 (94.1)	17 (100.0)	
Hemodialysis time (months); Mean ± SD	56.6±29.7	54.6±28.5	58.5±31.4	
Patient's initial weight; Mean ± SD	66.2±16.1	68.4±14.8	64.1±17.5	0.352^b^
Height; Mean ± SD	1.59±0.1	1.57±0.1	1.60±0.1	0.730^b^
BMI; Mean ± SD	26.3±6.4	27.6±6.2	25.0±6.5	0.101^b^
Blood type and Rh factor; n (%)				0.653^a^
A+	9 (26.5)	3 (17.6)	6 (35.3)	
B+	5 (14.2)	3 (17.6)	2 (11.8)	
AB+	1 (2.9)	1 (5.9)	0 (0.0)	
O+	18 (52.9)	9 (53.0)	9 (52.9)	
O-	1 (2.9)	1 (5.9)	0 (0.0)	

a: Fisher's exact test; b: Mann-Whitney test; Minimum Wage: approximately 250 US dollars (August 2022 - May 2023).

### Effect of the Intervention

The distribution of subjects in terms of laboratory and biochemical tests at baseline was comparable between CG and IG. Among the ten parameters evaluated, there were no statistically significant differences (*p>*0.005) in eight of them (Phosphorus, Potassium, Calcium, Pre-Hemodialysis Urea, Post-Hemodialysis Urea, URR, Creatinine, Kt/V) (Table 2). However, baseline comparison between CG and IG revealed significant differences in hematocrit (*p<*0.001) and hemoglobin (*p=*0.002).

At baseline, the mean hematocrit was 43.3±4.1 in CG and 37.1±5.0 in IG, indicating a significant group difference (*p<*0.001). Post-intervention, these values were 40.1±4.3 in CG and 37.6±4.4 in IG, with no significant difference observed (*p=*0.104). The prevalence of individuals developing anemia increased more in CG (from 17.6% to 29.4%) compared to IG (from 47% to 52.9%), a statistically significant difference (*p=*0.006). Similarly, baseline mean hemoglobin differed significantly between CG (13.2±1.3) and IG (11.6±1.6) (*p=*0.002). 

Post-intervention, values were comparable: CG (12.4±1.4) and IG (12.0±1.6), with no significant difference detected (*p=*0.373). However, there was a notable increase in the prevalence of altered hemoglobin in CG (23%) compared to a decrease in IG (11.8%), a statistically significant finding (*p=*0.017). Regarding calcium, 11.8% (n=4) of participants showed negative changes at baseline, while 100% (n=34) had no change post-intervention, indicating a significant difference (*p=*0.031). Mean calcium levels increased from 9.1±0.5 to 9.4±0.2 (*p<*0.001), remaining within the normal range. The post-intervention increase was statistically significant in both CG (*p=*0.015) and IG (*p=*0.013). Mean post-hemodialysis urea increased significantly from 32.4±14.1 to 39.0±19.9 (*p<*0.001), with 100% (n=34) of the sample experiencing a change. Between-group differences were significant for CG (*p=*0.002) and marginally non-significant for IG (*p=*0.057). Regarding URR, 29.4% (n=5) of CG participants showed a decrease, contrasting with 100% (n=17) of IG participants showing no change (*p=*0.04). URR decreased significantly from 73.3±7.4 to 69.7±12.9 (*p=*0.001), with the most pronounced reduction observed in CG compared to IG (*p=*0.003). Mean creatinine increased from 8.9±3.1 to 9.7±3.0 (*p=*0.002), with no significant between-group differences observed (*p=*0.006 for CG and *p=*0.09 for IG). Finally, there was a statistically significant decrease in mean Kt/V from 1.6±0.3 to 1.5±0.4 (*p=*0.001). This decline was driven by CG (from 1.6±0.4 to 1.4±0.5), while IG maintained Kt/V (1.6±0.3). The difference between CG and IG Kt/V post-intervention approached significance (*p=*0.056). The deterioration in dialysis quality in CG was significant (*p<*0.001), while the intervention effectively maintained dialysis quality without significantly altering this measure (*p=*0.521).

**Table 2:** Intergroup and intragroup comparison of laboratory and biochemical test results related to fluid volume and hemodialysis, before and after the intervention

Variables	Baseline			Outcome			Intragroup comparison		
	CG (n=17)	IG (n=17)	Intergroup *p-*value	CG (n=17)	IG (n=17)	Intergroup *p-*value	***p*-value**	***p*-value**	***p*-value**
Hematocrit			0.141^a^			0.296^a^	0.687^b^	1.000^e^	1.000^f^
Decreased	3 (17.6)	8 (47.1)		5 (29.4)	9 (52.9)				
Increased	1 (5.9)	0 (0.0)		0 (0.0)	0 (0.0)				
No change	13 (76.5)	9 (52.9)		12 (70.6)	8 (47.1)				
Mean	43.3±4.1	37.1±5.0	<0.001^c^	40.1±4.3	37.6±4.4	0.104^c^	0.106^d^	0.006^g^	0.523^h^
Hemoglobin			0.166^a^				0.508^b^	0.063^e^	0.625^f^
Decreased	7 (41.2)	12 (70.6)		12 (70.6)	10 (58.8)				
Increased	1 (55.9)	0 (0.0)		0 (0.0)	0 (0.0)				
No change	9 (52.9)	5 (29.4)		5 (29.4)	7 (41.2)				
Mean	13.2±1.3	11.6±1.6	0.002^c^	12.4±1.4	12.0±1.6	0.373^c^	0.338^d^	0.017^g^	0.320^h^
Phosphorus			1.000^a^			0.634^a^	1.000^b^	0.688^e^	1.000^f^
Decreased	1 (5.9)	1 (5.9)		0 (0.0)	2 (11.7)				
Increased	9 (52.9)	8 (47.1)		8 (47.1)	8 (47.1)				
No change	7 (41.2)	8 (47.0)		9 (52.9)	7 (41.2)				
Mean	4.8±1.2	4.4±1.4	0.409^c^	4.5±1.2	4.5±1.8	0.915^c^	0.918^d^	0.492^g^	0.619^h^
Potassium			1.000^a^			0.169^a^	0.774^b^	0.453^e^	1.000^f^
Decreased	1 (5.9)	1 (5.9)		1 (5.9)	0 (0.0)				
Increased	6 (35.3)	5 (29.4)		10 (58.8)	6 (35.3)				
No change	10 (58.8)	11 (64.7)		6 (35.3)	11 (64.7)				
Mean	5.0±0.4	4.9±0.8	0.500^c^	5.3±0.9	4.9±0.8	0.303^c^	0.113^d^	0.128^g^	0.380^h^
Calcium			0.537^a^			*	0.031	0.500^e^	0.125^f^
Decreased	2 (11.8)	2 (11.8)		0 (0.0)	0 (0.0)				
Increased	0 (0.0)	2 (11.8)		0 (0.0)	0 (0.0)				
No change	15 (88.2)	13 (76.4)		17 (100.0)	17 (100.0)				
Mean	9.0±0.4	9.1±0.6	0.352^c^	9.3±0.2	9.4±0.3	0.693^c^	<0.001^d^	0.015^g^	0.013^h^
Pre-HD Urea			1.000^a^			*	1.000^b^	1.000^e^	1.000^f^
Decreased	0 (0.0)	0 (0.0)		0 (0.0)	0 (0.0)				
Increased	17 (100.0)	16 (94.1)		17 (100.0)	17 (100.0)				
No change	0 (0.0)	1 (5.9)		0 (0.0)	0 (0.0)				
Mean	121.5±35.7	118.8±23.0	0.828^c^	128.9±33.5	128.4±28.5	0.923^c^	0.102^d^	0.344^g^	0.185^h^
Post-HD Urea			1.000^a^			0.398^a^	0.753^b^	0.375^e^	1.000^f^
Decreased	1 (2.9)	2 (11.8)		0 (0.0)	0 (0.0)				
Increased	1 (2.9)	1 (2.9)		5 (29.4)	2 (11.8)				
No change	15 (88.2)	14 (82.3)		12 (70.6)	15 (88.2)				
Mean	34.1±17.8	30.6±9.3	0.374^c^	43.1±26.1	34.8±10.1	0.191^c^	<0.001^d^	0.002^g^	0.057^h^
URR*			0.601^a^			0.044^a^	1.000^b^	0.500^e^	1.000^f^
Decreased	3 (17.7)	1 (5.9)		5 (29.4)	0 (0.0)				
Increased	0 (0.0)	0 (0.0)		0 (0.0)	0 (0.0)				
No change	14 (82.3)	16 (94.1)		12 (70.6)	17 (100.0)				
Mean	72.2±8.7	74.4±5.9	0.272^c^	66.6±16.9	72.8±5.8	0.102^c^	0.001^d^	0.003^g^	0.130^h^
Creatinine			*			*	1.000^b^	1.000^e^	1.000^f^
Decreased	0 (0.0)	0 (0.0)		0 (0.0)	0 (0.0)				
Increased	17 (100.0)	17 (100.0)		17 (100.0)	17 (100.0)				
No change	0 (0.0)	0 (0.0)		0 (0.0)	0 (0.0)				
Mean	9.5±3.1	8.3±3.0	0.324^c^	10.4±3.3	9.1±2.6	0.340^c^	0.002^d^	0.006^g^	0.093^h^
Kt/V***			0.227^a^			0.085^a^	0.125^b^	0.250^e^	1.000^f^
Unbalanced	2 (12.5)	0 (0.0)		5 (29.4)	1 (5.9)				
Balanced	14 (87.5)	17 (100.0)		11 (68.7)	16 (94.1)				
Mean	1.6±0.4	1.6±0.3	0.565^c^	1.4±0.5	1.6±0.3	0.056^c^	0.001^d^	<0.001^g^	0.521^h^

(a) Fisher's exact test for intergroup comparison; (b) McNemar's test for intragroup comparison; (c) Linear regression models were performed to adjust for group and furosemide use, given its potential role as a confounding variable; (d) Wilcoxon test for intragroup comparison; (e) McNemar's test for intragroup comparison in CG; (f) McNemar's test for intragroup comparison in the IG; (g) Wilcoxon test for intragroup comparison in the CG; (h) Wilcoxon test for intragroup comparison in the IG; *Calculation not possible; **URR: Urea Reduction Ratio; ***Dialysis adequacy measure.

## Discussion

Several blood components and clinical/laboratory markers are altered in CKD patients on hemodialysis.^16^ The major components that were altered in our study included the electrolytes calcium, phosphorus, potassium, creatinine, urea, and sodium, as well as red blood cells. Proper hemodialysis management and implementation of the intervention corrected or helped maintain these components within recommended serum concentrations, preventing hemodynamic instability and adverse human reactions. Regarding hematocrit and hemoglobin tests, both are interrelated and potentially impaired in the patients studied due to the organ's inability to produce erythropoietin.^17^ EFV in these patients decreases the concentration of these blood components, just as the loss of iron during dialysis impairs the synthesis of new red blood cells, leading to a decrease in these components.^18^

The intervention helped maintain hematocrit and hemoglobin levels in the IG, while there was a significant deterioration in the CG. This maintenance can be attributed to: 1) the nurse's signal to review the prescription of medications that stimulate the direct production of these blood components, which were prescribed for 85.3% of the sample; 2) the nutritional counseling; and 3) the understanding of the importance of maintaining self-care. This highlights the importance of the nurse's role in the management of these conditions. Implementation of the activities in this intervention also enabled early identification and targeted appropriate care in cases of anemia. By controlling intravascular fluid, the intervention helped prevent changes in hematocrit and serum hemoglobin parameters and associated complications, thereby improving patients' quality of life. Significant changes in electrolytes in general, and those altered by EFV in particular, can lead to adverse reactions in humans, depending on the ion. Regarding calcium, the intervention was effective in improving calcium levels in chronic kidney disease patients on hemodialysis in the IG tract. However, it should be considered that the routine intervention may have contributed to the serum improvement of this element in both groups, given the improvement in CG.

A correlation study found that the longer the time on hemodialysis, the lower the calcium concentration and the higher the final creatinine and potassium levels.^19^ Only 11.8% had lower calcium levels before the procedure, and none of the patients had hypocalcemia after the procedure, even those who had been on dialysis for 56.6±29.7 months. The correlation between hemodialysis time and calcium, creatinine and potassium levels, although weak, provides important insights into long-term management. This knowledge can help to personalize long-term treatment plans for hemodialysis patients. Educational efforts and medication adherence can be critical for electrolyte balance, which is essential for the prevention of bone and cardiovascular complications. It should be noted that altered and elevated creatinine levels were present in the entire sample before and after the intervention, as expected due to the physiological aspects of the disease, as well as a high prevalence of hyperphosphatemia. Hyperphosphatemia inhibits the enzyme renal 1-α-hydroxylase, which is responsible for converting vitamin D into its active form and impairs calcium absorption in the intestine.^20^ The joint analysis of electrolytes, such as calcium and phosphorus, highlights the importance of integrated thinking that takes into account the mutual influence of these elements. This approach can lead to a better understanding and management of electrolyte complications and prevent adverse reactions.

Urea, a waste product produced by the breakdown of proteins and eliminated from the blood by the kidneys^21^, was altered in nearly 100% of the pre- and post-hemodialysis samples. On the other hand, post-hemodialysis urea improved in IG and worsened in CG after the intervention, with significant results. The improvement in IG is related to the increase in the prevalence of patients who started to urinate after the intervention, with a statistical difference, and to the nutritional advice given for a balanced protein intake. The deterioration observed in the IG may be attributed to inadequate reinforcement of nutritional care and failure to improve uremia. Effective management of uremic control in EFV patients is complex and necessitates adherence to dietary guidelines akin to those for CKD, which advocate for a low-protein diet, restriction of processed foods, and increased fiber intake.^22^ Research indicates that these dietary interventions effectively mitigate the accumulation of uremic toxins.^23^

Creatinine, by the same principle as urea, also accumulates in the body of this population.^24^ In the present study, it remained altered in all participants, even after the intervention. This result was expected, as persistently elevated serum creatinine is a diagnostic and prognostic feature of CKD itself.^25^ Dehydration can elevate the body's creatinine concentration.^26^ However, this possibility was excluded since there was no deterioration in assessments of "skin turgor", "mucosal moisture", or "eye appearance", indicating that these patients maintained adequate hydration. Considering the worsening of urea, URR, and creatinine values, along with the increase in average calcium and phosphorus levels within recommended ranges, it is hypothesized that the elevated organic compound levels in the IG reflect the impact of nutritional counseling, a key component of the intervention. This likely contributed to improved nutritional status and consequent increases in nitrogen compounds within this group. In contrast, these changes were not observed in the CG, where phosphorus levels decreased within the recommended range, and nutritional counseling was not provided as part of the intervention. These findings underscore the importance of nurses acquiring specialized nutrition knowledge for effective patient monitoring.

The mean values of URR and Kt/V in hemodialysis patients serve as critical indicators of dialysis adequacy in chronic kidney disease, guiding multidisciplinary care tailored to patient needs.^9^ Normal thresholds for Kt/V are typically above 1.20, and for URR, above 65%.^27^ In a recent cross-sectional study of 100 hemodialysis patients, the average URR was 25.24±15.59, and Kt/V was 0.73±0.162.^9^ Similarly, our study found a baseline URR of 73.3±7.4, which decreased to 69.7±12.9 post-intervention, primarily due to deterioration observed in the CG. In contrast, the IG maintained adequate URR within the 70% range (from 74.4±5.9 to 72.8±5.8 post-intervention).

As for Kt/V, this study showed a significant deterioration in CG, from 1.6±0.4 to 1.4±0.5, while in IG the average remained at 1.6±0.3, although one patient in this group had an unbalanced Kt/V. For URR, the patients in IG had the same average dialysis quality, while those in CG were close to the 65% cut-off, indicating a deterioration in hemodialysis adequacy. Similarly, CG had the worst performance in dialysis quality during the 12 hemodialysis sessions after the intervention date. The literature does not indicate whether there is a difference in dialysis adequacy between patients using an arteriovenous fistula and those using a hemodialysis catheter. The statistically significant differences for both measures of dialysis adequacy may be related to the performance of the intervention, which eliminated the presence of EFV in 35.3% of the IG sample. The persistence of hypervolemia in 94.1% of the CG may justify the marked deterioration in the quality of hemodialysis in these patients, since EFV directly affects the quality of dialysis in patients with chronic kidney disease.^28^ Hypervolemia directly affects the quality of dialysis, and its elimination contributed to more effective dialysis.

The difference between the mean results for URR, urea reduction rate considering its pre-HD and post-HD values, and Kt/V, validate the intervention and show that its implementation had an influence on improving the quality of dialysis in the IG. It should be noted that all patients in the dialysis clinic used the same brand of dialyzer, prescribed appropriately according to the client's weight and clinical conditions.^29^. The maintenance and improvement of dialysis adequacy measures in the IG after the intervention highlights the effectiveness of the practices implemented and reinforces the need for nursing strategies focused on resolving hypervolemia and nutritional education to optimize hemodialysis outcomes. It is suggested that a multiprofessional approach with the active participation of nurses, nutritionists and physicians is fundamental to improve the quality of dialysis and, consequently, the clinical outcomes of patients.

Nursing interventions targeting these patients have the potential to improve their quality of life and health.^30^ Combining interventions targeting electrolyte imbalance with this tested intervention could potentially be beneficial in resolving the EFV and electrolyte imbalance common in these patients.^31^^-^^33^ It highlights the potential of nursing practices to improve the health and quality of life of CKD patients on hemodialysis, supporting the adoption of similar interventions in other clinics. This study provides new evidence on the effectiveness of nursing interventions in the management of CKD patients on hemodialysis. By focusing on an integrated approach that includes medication review, nutritional counseling and fluid control, the study demonstrates that structured interventions can significantly improve laboratory parameters and dialysis adequacy. This not only improves patients' quality of life, but also reduces the risk of complications related to electrolyte imbalances and dialysis inadequacy.

With the positive impact of the intervention evident in stable hemodialysis patients, as indicated by stable or improved post-intervention test results without significant fluctuations, it is clear that this intervention could effectively address electrolyte and blood component disorders. However, caution is advised regarding the risk of electrolyte imbalances, given their high prevalence in this patient population. Experts emphasize the importance of publishing research on laboratory parameters and associated risk factors in hemodialysis patients to ensure dialysis adequacy and early detection of CKD-related complications.^13^ Furthermore, future studies should explore implementing this intervention to evaluate its impact on the quality of life among CKD patients undergoing hemodialysis, considering the notable improvements observed in biochemical profiles, laboratory measures, and dialysis adequacy in patients who received the intervention.

Strength and limitations. One of the limitations of the study was the inability to calculate patients' GFR for comparison within and between groups. Other tests, such as serum glucose, were not analyzed because not all patients underwent the same tests in addition to the routine tests, which are prescribed only when necessary, making further comparisons impossible. It should be emphasized that the laboratory test results were provided by the dialysis center itself and it was not possible to include other blood components. The exclusion of additional non-standard tests reflects a realistic and practical approach and is taken into account in randomized clinical trials to ensure that the results analyzed are clinically relevant.

Implications for clinical practice. The results of this research provide a significant contribution to nursing practice in the care of patients with end-stage CKD in a real-world healthcare setting. By analyzing biochemical test results and dialysis adequacy measures, nurses can identify early clinical changes and variations in electrolyte levels and other laboratory tests, allowing timely implementation of the evaluated intervention to mitigate complications and improve dialysis adequacy measures. Applying this intervention not only improves test results, but also improves patient quality of life while reducing the risk of adverse events related to fluid and electrolyte imbalances in this population. This proactive, evidence-based approach by nurses plays a key role in promoting effective care for CKD patients with excess fluid volume, inadequate fluid control and electrolyte disturbances, as well as improving dialysis adequacy. This underscores the critical importance of the nurse's role in identifying, preventing and managing these complex conditions.

Conclusion. The intervention studied was effective in improving therapy outcomes, particularly in terms of fluid volume and hemodialysis, in patients with end-stage CKD who initially had excess fluid volume. The statistically significant results indicated significant improvements in laboratory tests, dialysis adequacy and patient care process. The statistical difference between the groups was remarkable for the mean values of calcium, post-hemodialysis urea and creatinine, demonstrating the direct impact of the intervention. In addition, there were statistically significant improvements in overall dialysis quality, as evidenced by the between-group mean URR and Kt/V. This highlights the importance of taxonomies and targeted care plans for patients with kidney disease and chronic hemodialysis.
